# Utility of unenhanced postmortem computed tomography for investigation of in-hospital nontraumatic death in children up to 3 years of age at a single Japanese tertiary care hospital

**DOI:** 10.1097/MD.0000000000020130

**Published:** 2020-05-08

**Authors:** Masanori Ishida, Wataru Gonoi, Go Shirota, Hiroyuki Abe, Yukako Shintani-Domoto, Masako Ikemura, Tetsuo Ushiku, Osamu Abe

**Affiliations:** a Department of Radiology; b Department of Pathology, Graduate School of Medicine, The University of Tokyo, Bunkyo-ku, Tokyo, Japan.

**Keywords:** autopsy, baby, children, computed tomography, infant, postmortem imaging

## Abstract

To evaluate the utility of unenhanced postmortem computed tomography (PMCT) for the investigation of in-hospital nontraumatic death in children up to 3 years of age.

This study included the cadavers of children who died from intrinsic diseases before 3 years of age. The major underlying disease and the main organ–disease systems associated with the immediate causes of death were determined by clinical evaluation, PMCT, and autopsy, which were used as a reference standard. The rates of concordance between the former two methods and autopsy were calculated for all cases.

In total, 22 cadavers (12 male and 10 female; mean age, 6.1 ± 8.2 months) were included. The rates of concordance between clinical evaluation/PMCT and autopsy for diagnosis of the major underlying disease and main organ–disease systems associated with the immediate causes of death were 100%/36% (*P* = .0015) and 59%/41% (*P* = .37), respectively. In cases where the respiratory system was associated with the immediate cause of death, PMCT showed greater diagnostic sensitivity (90%) than did clinical evaluation (20%). In contrast, the diagnostic sensitivity of PMCT was lower than that of clinical evaluation in cases involving disorders of the cardiac system and multiple organ systems (0% vs 100% for both).

The findings of this study suggest that the use of unenhanced PMCT with clinical evaluation can result in improved detection of the immediate cause of death in select cases of in-hospital nontraumatic death before 3 years of age.

## 1 Introduction

Postmortem computed tomography (PMCT) is increasingly being used as an adjunct to conventional autopsy in postmortem studies, with a worldwide decrease in the autopsy rate.^[1–3]^ For a wide age group, computed tomography (CT) plays a certain role in postmortem investigations for diagnosing the cause of death. The use of PMCT is considered to improve the quality of postmortem investigations.^[2,4]^ In Japan, unenhanced CT is the standard tool for postmortem imaging because of its rapid scanning speed and convenience.^[5]^ Unenhanced PMCT is a noninvasive imaging technique that is rapidly and easily performed, and a bereaved family may accept this examination without much resistance.

Several researchers have reported the postmortem changes observed on PMCT,^[6–12]^ while some studies have assessed the usefulness of PMCT for vascular, cardiac, and bone imaging in fetuses and children.^[13–15]^ However, in children, unenhanced PMCT is considered to hold limited value because the size of the body or organs is smaller and the imaging resolution is low. Although it was found that contrast-enhanced PMCT is required to assess the structures in detail, this technique is slightly invasive and seldom performed at institutions other than those specialized in postmortem investigations.

During the course of daily clinical practice, where several cases of nontraumatic in-hospital deaths are encountered, we found that the use of unenhanced PMCT with consecutive autopsy as a reference standard does add some value for determining the cause of death in children. The significance of PMCT in pediatric postmortem investigations is thought to differ according to age and the presence or absence of trauma, considering the causes of death vary among age groups, particularly in children. In Japan, congenital diseases, including malformations and chromosomal abnormalities, are the leading causes of death until the age of 3 years, while the causes of death differ for older age groups.^[16]^

Assessment of the utility of unenhanced PMCT as an adjunct to conventional autopsy for pediatric cases is important for improving the quality of postmortem investigations. However, only a few studies have assessed the value of unenhanced PMCT relative to that of autopsy for pediatric cases of in-hospital deaths.^[17,18]^ Therefore, the aim of the present study was to evaluate the utility of unenhanced PMCT for investigating in-hospital nontraumatic death in children up to 3 years of age by comparing its findings with those of clinical evaluation, using autopsy as the reference standard. To the best of our knowledge, this is the first study to compare clinical evaluation, PMCT, and autopsy for in-hospital pediatric deaths.

## 2 Materials and methods

### 2.1 Subjects

This prospective study (Ethical Committee N° 2076-[12]) was approved by the Ethical Committee of the University of Tokyo Hospital on June 9, 2008, and informed consent for use of the cadavers in our research was obtained from the families of the deceased. We performed PMCT and conventional autopsy for the cadavers of pediatric patients who had died from nontraumatic causes in our academic tertiary care hospital between April 2009 and December 2018. Children who died before 3 years of age were included by considering the distribution of causes of pediatric death in Japan.^[19]^ In this age group, congenital malformations and deformities, chromosomal abnormalities, malignant neoplasms, heart diseases, and respiratory diseases are the main causes of death in Japan. Stillborn babies and cases of trauma-related death were excluded. All cadavers were placed in the supine position at room temperature from the time of death until PMCT examination.

### 2.2 PMCT technique

All PMCT studies were performed without contrast enhancement using a four-detector row CT scanner (Robusto, Hitachi Medical Corporation, Tokyo, Japan) in the helical mode in the craniocaudal direction. The cadaver was placed in the supine position with the arms at the sides during imaging, and the area between the head and toes was scanned. The scan parameters were as follows: slice thickness, 1.25 mm; slice interval, 1.25 mm; rotation time, 0.5 s; tube voltage, 120 kVp; and tube current, 250 mA. Image reconstruction was performed with a 1.25-mm thickness, 350-mm field of view, and 512 × 512 image matrix.

### 2.3 Autopsy technique

All autopsies were performed by board-certified pathologists immediately after PMCT. The pathologists were informed about the patients’ clinical histories and details regarding the circumstances of death, although they were unaware of the PMCT findings documented by the radiologists. Autopsy was performed using conventional procedures. Where possible or when necessary, brain autopsy was performed. Any pathological lesions in the entire body were noted. The pathologists diagnosed the main pathological lesions, that is, the major underlying disease, and estimated the immediate cause of death on the basis of their own findings and relevant clinical findings.

### 2.4 PMCT image evaluation

**Figure 1:**
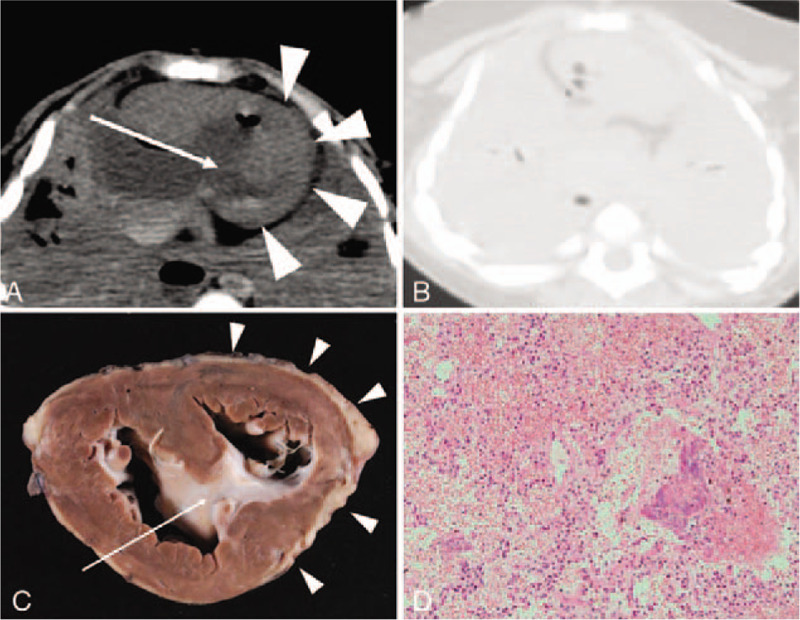
Representative case involving a deceased boy aged 1 year and 3 months. Computed tomography (CT) was performed 16 h and 54 min after death. (A) Ventricular septal defect (arrow) and left ventricular hypoplasia (arrowheads) are suspected on postmortem CT (PMCT). (B) Bilateral lung aeration is not seen on PMCT. Therefore, the main organ–disease system associated with the immediate cause of death was suspected to be the respiratory system. (C) Ventricular septal defect (arrow) and left ventricular hypoplasia (arrowheads) are confirmed by sequential autopsy. Compensatory hypertrophy and dilated hypertrophy of the right ventricle are also seen. Respiratory failure caused by pneumonia and pulmonary edema was confirmed as the immediate cause of death. (D) Histopathological analysis (hematoxylin and eosin staining) shows inflammatory cell infiltration and lung congestion.

The PMCT images were analyzed using Image J 1.46r (Wayne Rasband, NIH, Bethesda, MD), which was downloaded at http://imagej.nih.gov/ij. Using two-dimensional axial datasets, three board-certified radiologists with 10, 9, and 8 years of experience in postmortem imaging, respectively, interpreted the images after referring to the patients’ medical histories. The major underlying disease that could result in individual mortality was identified on the images (Fig. 1A). After diagnosis of the major underlying disease related to death, the most probable culprit lesion or abnormality that played a role in the patient's death was estimated and the main organ–disease systems associated with the immediate causes of death were recorded (Fig. 1B).

### 2.5 Concordance among clinical evaluation, PMCT, and autopsy

The clinical diagnoses recorded by physicians before and at the time of death were obtained from clinical records prepared in the hospital. A review of the clinical history of patients included a summary of the relevant antemortem information and findings of physical examination, antemortem imaging studies (i.e., ultrasonography, CT, and magnetic resonance imaging), and antemortem blood tests. The major underlying disease identified by clinical evaluation, PMCT, and autopsy was noted. Subsequently, the main organ–disease systems associated with the immediate causes of death were identified as follows: neurological, cardiac, respiratory, abdominal, musculoskeletal, and multiple organ systems, as modified from previous studies.^[4,20]^ If the lesion that resulted in death could not be determined or was unclear, it was categorized as “undetermined.” The diagnosis established at the pathologists’ discretion and by consensus during autopsy was considered the gold standard (Fig. 1C and D). The primary outcome was the concordance between clinical evaluation/PMCT and autopsy in terms of diagnosis of the major underlying disease. The secondary outcome was concordance in terms of identification of the main organ–disease systems associated with the immediate causes of death. The concordance rates were obtained by calculating the number of cases where the diagnoses established by clinical evaluation/PMCT matched the diagnosis confirmed by autopsy, and the values are expressed as percentages. All statistical analyses were performed using R, version 3.5.3 (The R Foundation for Statistical Computing, Vienna, Austria; http://www.r-project.org/), and the obtained data were analyzed using Fisher's exact test. A *P*-value of <.05 was considered statistically significant.

## 3 Results

### 3.1 Subjects

The final study population comprised 22 cadavers (12 male and 10 female; age, 6 h to 2 years and 5 months; mean age, 6.1 ± 8.2 months). Cardiopulmonary resuscitation (including artificial respiration, chest compression, and infusion) had been performed for five of the 22 children. PMCT was performed ∼2 to 17.5 h (mean, 9.2 ± 4.8 h) after death, while autopsy was performed ∼2 to 17.5 h (mean, 1.1 ± 0.6 h) after PMCT. Brain autopsy was performed for seven subjects.

### 3.2 Major underlying disease

**Figure 2:**
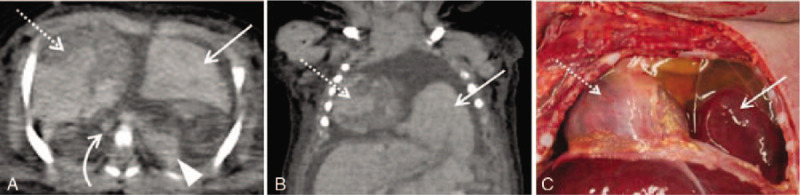
Representative case involving a deceased boy aged 1 day. Computed tomography (CT) was performed 17 h and 25 min after death. (A) Postmortem CT (PMCT) shows that the left thoracic cavity contains multiple organs such as the lateral segment of the left hepatic lobe (arrow), spleen (arrowhead), and a part of the intestine. The heart (dotted arrow) and thoracic aorta (curved arrow) are shifted to the right. (B) A coronal multiplanar reconstruction image proves useful for determining the orientation of the organs (arrow, lateral segment of the left hepatic lobe; dotted arrow, heart). Left congenital diaphragmatic hernia was suspected as a major pathological abnormality. Respiratory failure was considered the immediate cause of death. (C) Autopsy reveals left congenital diaphragmatic hernia and herniated structures, including the lateral segment of the left hepatic lobe (arrow) and heart (dotted arrow). Respiratory failure was confirmed as the immediate cause of death.

**Figure 3:**
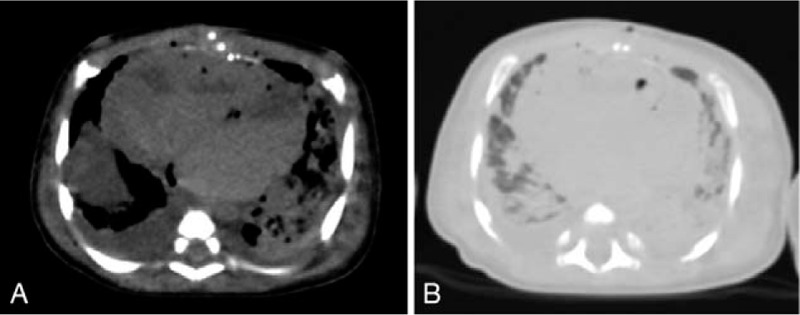
A representative case involving a deceased boy aged 1 month. Computed tomography (CT) was performed 12 h and 11 min after death. (A) A single ventricle and single atrium are suspected on postmortem CT (PMCT). Intracardiac gas due to the resuscitation attempt is also found. (B) Bilateral pulmonary consolidation is observed on PMCT, and the lungs were suspected to be the main organs associated with the immediate cause of death. However, autopsy localized the heart as the main organ associated with the immediate cause of death, which was confirmed to be circulatory failure.

Clinical evaluation and autopsy diagnosed the same major underlying disease in all 22 subjects; the diagnoses included double outlet right ventricle (four cases); hypoplastic left heart syndrome and cardiomyopathy (three cases each); congenital diaphragmatic hernia and a single ventricle and atrium (two cases each); and mediastinal tumor, Dandy–Walker malformation, myelomeningocele, acute myeloid leukemia, pyruvate dehydrogenase complex deficiency, trisomy 18, pulmonary atresia, and transposition of the great arteries (one case each). In contrast, PMCT and autopsy diagnosed the same major underlying disease in only eight subjects; the diagnoses included congenital diaphragmatic hernia (two cases; Fig. 2A–C) and hypoplastic left heart syndrome, double outlet right ventricle, a single ventricle and atrium (Fig. 3A), mediastinal tumor, Dandy–Walker malformation, and myelomeningocele (one case each). Thus, the rate of concordance between clinical evaluation and autopsy was significantly higher than that between PMCT and autopsy (Table 1).

**Table 1:**
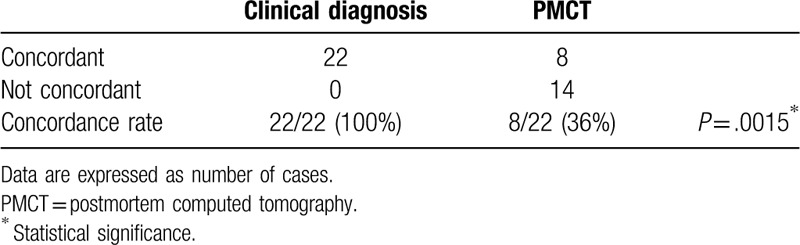
Concordance of clinical diagnosis and postmortem computed tomography with autopsy in terms of the major underlying disease in pediatric cases of in-hospital nontraumatic death.

### 3.3 Main organ–disease systems associated with the immediate causes of death

The rates of concordance between PMCT/clinical evaluation and autopsy in terms of the main organ–disease systems associated with the immediate causes of death are presented in Table 2. The rate of concordance between clinical evaluation and autopsy was slightly higher than that between PMCT and autopsy (59% vs 41%, *P* = .37). Compared with clinical evaluation, PMCT was significantly more sensitive in diagnosing respiratory disorders (90% vs 20%; *P* = .005) and significantly less sensitive in diagnosing cardiac disorders (0% vs 100%; *P* < .001; Fig. 3B). Although clinical evaluation (100%) was more effective than PMCT (0%) in diagnosing multiple organ disorders, the difference was not significant (*P* = .1).

**Table 2:**
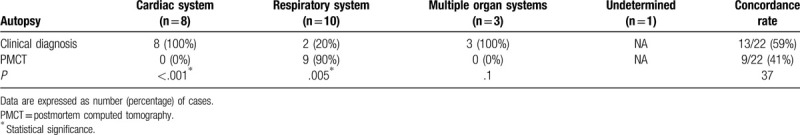
Concordance of clinical diagnosis and postmortem computed tomography with autopsy in terms of the main organ–disease systems associated with the immediate causes of death in pediatric cases of in-hospital nontraumatic death.

### 3.4 Concordance rates according to the patient's age

According to epidemiological data regarding causes of death in pediatric patients under 3 years of age, we divided our patients into 0 to 1-year and 1 to 3-year age groups.^[16]^ Concordance rates for identification of the main organ–disease systems associated with the immediate causes of death in the two groups are shown in Table 3; the rate was significantly higher for clinical evaluation than for PMCT in the 0 to 1-year age group.

**Table 3:**
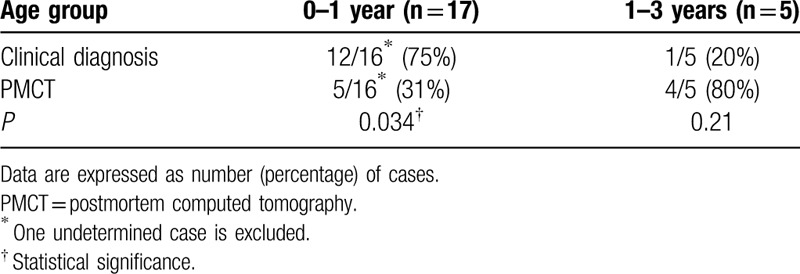
Concordance of clinical diagnosis and postmortem computed tomography with autopsy in terms of the main organ–disease systems associated with the immediate causes of death in age-stratified pediatric cases of in-hospital nontraumatic death.

## 4 Discussion

In the present study, we assessed the concordance of clinical evaluation and PMCT with autopsy in terms of identification of the major underlying disease and main organ–disease systems associated with the immediate causes of natural in-hospital death in infants and toddlers. The rates of concordance between clinical evaluation/PMCT and autopsy for diagnosis of the major underlying disease and main organ–disease systems associated with the immediate causes of death were 100%/36% and 59%/41%, respectively. In cases where the respiratory system was associated with the immediate cause of death, PMCT showed greater diagnostic sensitivity (90%) than did clinical evaluation (20%). In contrast, the diagnostic sensitivity of PMCT was lower than that of clinical evaluation in cases involving disorders of the cardiac system and multiple organ systems (0% vs 100% for both).

Postmortem imaging for babies and children is important to confirm the suspected antemortem abnormalities and the presence of any other abnormality that is not detected during antemortem examination.^[15,21]^ Furthermore, one of the important applications of pediatric postmortem imaging is investigation of additional information such as the cause of death and provision of information that complements the details noted by conventional autopsy.

In the present study, unenhanced PMCT was ineffective in estimating the major underlying disease present before death, whereas clinical evaluation could diagnose all major underlying diseases that were confirmed by autopsy. These findings are similar to those of previous studies involving adult cases of in-hospital death.^[22,23]^ For pediatric patients, however, the accuracy of clinical evaluation for diagnosis of the major underlying disease has been reported to be no more than 50%.^[24–26]^ Therefore, the 100% concordance rate obtained for clinical evaluation and autopsy in the present study may have been affected by population bias. Nevertheless, we found that PMCT was poorer than clinical evaluation in estimating the major underlying disease and inadequate for determining the pathological conditions present before death in most children; this highlights the importance of autopsy.

With regard to identification of the main organ–disease systems associated with the immediate causes of death, both clinical evaluation and PMCT showed moderate concordance with autopsy, with PMCT proving slightly less sensitive (41%) than clinical evaluation (59%). Major gross pathologies can be occasionally detected by unenhanced PMCT, although the sensitivity of PMCT is lower than that of autopsy.^[27]^ In the present study, clinical evaluation was less sensitive than PMCT in diagnosing respiratory system disorders related to death, probably because pathological changes in the lung rapidly occur in the agonal stage in many cases,^[28,29]^ resulting in overlooks during clinical evaluation. Such lung changes can be detected by PMCT because it is performed after death. Costache et al reported that pulmonary pathologies were the most frequently overlooked pathologies by clinicians evaluating newborns.^[25]^ This supports the fact that only a few fatal lung lesions were detected by clinical evaluation in the present study. In contrast, clinical evaluation was effective in detecting fatal cardiac lesions because various antemortem examinations were performed and the major disease was already diagnosed. PMCT could not diagnose eight fatal cardiac lesions that were revealed during autopsy. It should be noted that PMCT has little value for detecting major pathological lesions in infants. This is particularly true for congenital cardiac disease, which exhibits complex anatomical abnormalities that are difficult to detect. However, congenital abnormalities in the chest, excluding the heart, were partly evident on PMCT images obtained before autopsy. For example, a thoracic hernia could be observed, and the orientation of the thoracic structures was easily understood. This noninvasive feature is one of the advantages of PMCT. We also found that PMCT was less sensitive than clinical evaluation in identifying multisystem disorders associated with death, probably because such disorders present no or small morphological abnormalities that are difficult to evaluate on PMCT.

Nevertheless, PMCT can explain the situation around the time of death or the immediate cause of death in infants and children.^[30–33]^ Proisy et al used autopsy as a reference standard and found that sudden unexpected and nontraumatic deaths in infancy were correctly explained by PMCT in 15 of 47 cases (32%),^[30]^ while Noda et al reported that PMCT identified the cause of death confirmed by autopsy in 57% infants and children who died from intrinsic causes.^[31]^ The present study determined a 41% concordance rate for PMCT and autopsy in terms of identification of the main organ–disease systems associated with the immediate causes of death; this was within the range of all previous results. With regard to the cause of unnatural deaths in pediatric patients, Sieswerda-Hoogendoorn et al reported a concordance rate of ∼70% to 80% for PMCT and autopsy. Moreover, the diagnostic accuracy of PMCT was significantly lower in cases of natural death than in cases of unnatural death.^[32]^ We also found that clinical evaluation and autopsy identified the same organ–disease systems associated with the immediate causes of death in only 59% cases. Thus, both clinical evaluation and PMCT were inadequate for establishing the cause of death in infants and toddlers aged <3 years. This highlights the importance of autopsy as a reference standard, as previously mentioned by Sonnemans et al.^[33]^ Gordijn et al found that autopsy revealed a change in diagnosis or additional findings^[24]^; similarly, autopsy findings were different from those of clinical evaluation and PMCT in several cases in the present study.

With regard to age, PMCT correctly identified the main organ–disease systems associated with the immediate causes of death in four of five children aged 1 to 3 years; this could not be achieved by clinical evaluation. However, the sensitivity of clinical evaluation in the 0 to 1-year age group was relatively good and higher than that of PMCT. The rate of concordance between PMCT and autopsy was higher in the 1 to 3-year age group than in the 0 to 1-year age group. The increased spatial resolution of CT with the increase in the child's growth could be one of the reasons for this finding.

This study has some limitations. First, the sample was too small, and the spectrum of diagnoses was varied. Therefore, it is somewhat difficult to generalize the findings. Inclusion of more subjects could have yielded statistical significance in some comparisons. It is hoped that long-term longitudinal studies at multiple institutions will be conducted. Second, brain autopsy was not performed in all cases. However, there was no subject with a fatal intracranial lesion estimated by clinical and PMCT evaluations, and we believe that our autopsy findings were not affected by the performance of brain autopsy. Third, there were many cases with congenital heart defects in the study. This bias may be associated with the patient population at our academic tertiary care hospital. Fourth, antemortem rehydration may promote consolidation of the lung parenchyma, which complicates the differentiation of an antemortem pathological condition from a postmortem change.

In conclusion, the findings of this study suggest that the use of unenhanced PMCT with clinical evaluation can result in improved detection of the immediate cause of death in select cases of in-hospital nontraumatic death before 3 years of age.

## Author contributions

**Conception:** Masanori Ishida.

**Data acquisition:** Masanori Ishida, Wataru Gonoi, Go Shirota, Hiroyuki Abe, Yukako Shintani-Domoto, Masako Ikemura.

**Analysis:** Masanori Ishida.

**Drafting the work:** Masanori Ishida.

**Revising:** Wataru Gonoi, Go Shirota, Hiroyuki Abe, Yukako Shintani-Domoto, Masako Ikemura, Tetsuo Ushiku, Osamu Abe.

**Supervision:** Tetsuo Ushiku, Osamu Abe.
